# Which is the best way to treat a stone on a flexible ureterorrenoscopy? | *Opinion: Dusting*

**DOI:** 10.1590/S1677-5538.IBJU.2017.05.03

**Published:** 2017

**Authors:** Antonio Correa Lopes

**Affiliations:** 1 Grupo de Litíase e Endourologia da Disciplina de Urologia da Faculdade de Medicina ABC, Santo André, SP, Brasil

**Keywords:** Calculi, Lasers, Solid-State, Kidney

Flexible ureterorenolithotripsy is rapidly developing and becoming the treatment of choice around the World for the invasive treatment of lithiasis (1). According to calculi dimensions, they can be removed integrated or by using an intracorporeal lithotripter.

According to laser parameters adjustments, it is possible to vaporize calculi (“dusting”). In this case, it is necessary to use high frequency of impulses (>15HZ), low energy (<0.5J) and long pulses (800 ^ɰ^sec), whenever the equipment allows for these options.

Recently, it has been debated which is the best way to program the equipment at the moment of calculi lithotripsy. There are few evidences in literature to conclude, but it seems that dusting has some advantages in relation to fragmentation + basketing.

An experimental work performed in 1999 showed that the use of high energy during lithotripsy increased the quantity of larger fragments; the authors suggested the use of <1 J energy and high frequency of impulses, that characterizes dusting (2). Another more recent experimental study also verified that, by using several potencies and frequencies, that the produced fragments were lower than 1 mm in almost all groups that used 0.2J of energy. Those fragments increased proportionally to the increase of Joules of Holmium laser (3). At present, endourologic post-surgical residual calculi have become a future concern, since they can cause pain, urinary tract obstruction and need of reintervention. The EDGE group collected data from 6 centers and verified that among 232 patients with residual lithiasis, 44% evolved with some colic event and 29% needed surgical reintervention. Fragments bigger than 4 mm were the most probable to grow (p<0.001) and associated with bigger complications (p=0.039) (4). Also, when bigger fragments are produced, the need for basket removal generates more movement of the appliance through the sheath, increasing the risk of displacement and ureteral lesions.

It is possible to treat efficiently calculi generating power or very tiny fragments that don't need to be removed, dismissing the need of a basket and lowering costs (3). Morhardt, using a 120 W Holmium Laser and dusting, showed efficient resolution of renal and ureteral bigger calculi (1.5-1.7cm) (5). A prospective multicenter study (EDGE group) compared dusting method to fragmentation and basketing in the treatment of intra-renal calculi with 5 to 20mm. 59 patients were studied. Calculi were bigger in the dusting group and stone-free rate was lower (60.9%). But only 17% of residual fragments were bigger than 4mm and there was no difference in relation to reintervention, hospital readmission or pain. Dusting was favored by shorter surgical time and only 27.5% of procedures used sheath (6). Glick-man analyzed laser adjustments during ureterorenolithotripsy in 103 patients. One group used dusting adjustment with low energy (0.2-0.4J) and high frequency (50HZ) and another used higher energy (0.5-1J) with frequency from 5 to 10HZ. In only 3.8% of dusting cases it was inefficient and it was necessary to change to higher energy. The authors concluded the procedure is efficient, with finer fragments and without the need of basketing (7).

In the specific treatment of ureteral calculi, the use of lower energy during dusting reduces the incidence of retropulsion of fragments and eventual return to the intra-renal collector system. Several studies showed that this situation is more frequent when it is used higher energy of laser (2, 3, 7-9). In an experimental model, retropulsion of ureteral calculi was higher and faster when it was used 0.6J/5Hz. With these parameters, 100% of calculi had retropulsion in less than 3 minutes of fragmentation. Fragmentation with 0.2J/15HZ (dusting) caused 60% of retropulsion, but only after 10 minutes of exposure of calculi to impulses, allowing for the resolution of calculi before retropulsion. In a longer time, it was possible to fragment bigger calculi (10). When it was used higher energy, anti-retropulsion devices increased the efficiency of lithotripsy (3), but it must be considered that the need of another material increases the costs. When Ho Yag laser equipment allows for change of pulse length, retropulsion is lower when a longer pulse is used, resulting in better results, since the calculus remains immobile for longer period under fragmentation (11, 12).

Another advantage observed in a comparative work was that dusting fragmentation preserved more the laser fiber: the need to trim the tip during the procedure was less frequent when this adjustment was used (6). An in vitro experiment using holmium laser and dusting showed that the use of longer pulses also generates less degradation of the fiber tip (12).

Dusting, a recent method with few studies and strong evidences, has several advantages in relation to fragmentation and basketing. In summary, the main benefits are:

1. Generation of powder or little fragments, minimizing the risk of recurrence and future complications;
2. Lower costs, since it does not use baskets and spares the laser fiber, with less degradation of its tip. Also, there is no need to extract fragments and it is possible not to use the ureteral sheath, reducing even more the costs.
3. Lower retropulsion of ureteral calculi, reducing the risk of migration to the kidney and inefficiency of the procedure.

I believe that dusting must be the initial choice for intracorporeal lithotripsy. In case of failure during the procedure, it is possible to increase energy, reduce frequency and pulse length, modifying the method to fragmentation and basketing.
